# Muscles Flexed, Minds Relaxed: Investigating the association between strength training and anxiety

**DOI:** 10.1192/j.eurpsy.2025.1029

**Published:** 2025-08-26

**Authors:** S. Ben Aissa, C. Najar, K. Razki, M. Cheour, F. Fekih-Romdhane

**Affiliations:** 1 Razi Hospital, La Manouba; 2 Faculty of Medicine of Tunis, Tunis El Manar University, Tunis, Tunisia

## Abstract

**Introduction:**

Strength training approaches have seen a significant increase in popularity in the fitness and wellness industries in recent years. This rise is fueled not only by the desire for physical prowess and muscle development, but also by a growing awareness of its potential impact on mental health. While the traditional focus of research on strength training has primarily been on its physical benefits, there exists a developing enthusiasm within the scientific community to explore deeper into its implications for mental health, particularly with anxiety levels.

**Objectives:**

The purpose of this study is to examine the link between strength training intensity and anxiety levels among active gym-goers in Tunisia.

**Methods:**

This is a cross-sectional study, conducted from February to March 2024. Participants were recruited online through social media platforms ( Tunisian facebook groups and fitness forums) using a posted survey link. We’ve included respondents who are 18 years of age or older who have been active in strength training with a gym membership for 1 month or more. The respondents were required to answer a questionnaire that included socio-demographic questions and to provide strength training intensity related details ( sessions frequency, duration, perceived overall intensity using likert scale). Anxiety levels were evaluated using the Generalized Anxiety Disorder 7 (GAD-7) scale.

**Results:**

The overall number of participants was 72, with 86% being male. The majority of responders (n=65, 90.2%) indicated that they performed strength training exercises at least three times per week, with an average session length of 45 minutes. In terms of strength training intensity, 38.8% (n= 28) of participants reported high-intensity sessions, 48.6%(n=35) moderate-intensity sessions, and the remaining participants reported low-intensity sessions.

Analysis showed a mean anxiety score of 6.1 (SD = 3.8) on the GAD-7 scale, indicating absent to mild anxiety symptoms among respondents.

Further analysis revealed a negative association between strength training intensity and anxiety levels (r = -0.59, p = 0.026), implying that higher intensity sessions were linked with lower anxiety scores.

**Conclusions:**

In conclusion, this study provides insight into the level of anxiety of Tunisian gym-goers who participate in strength training, emphasising the potential of exercise interventions to improve mental health in this society. Moving forward, these findings can help to shape targeted interventions and wellness efforts, promoting holistic approaches to well-being that prioritise both physical and psychological health.

**Disclosure of Interest:**

None Declared

